# Grammaticalization of discourse markers: views from Jordanian Arabic

**DOI:** 10.1016/j.heliyon.2021.e07632

**Published:** 2021-07-22

**Authors:** Abdulazeez Jaradat

**Affiliations:** Applied Science Private University, Amman, Jordan

**Keywords:** Grammaticalization, Discourse marker, Discourse linker, Sentence grammar, Peripheral grammar, Jordanian Arabic

## Abstract

The inclusion of discourse markers (DMs), which are commonly depicted as syntactically non-integrated elements, within the domain of grammaticalization has been a controversial topic in the last two decades. The emergence of DMs for some researchers are cases of grammaticalization, whereas they are viewed by some other researchers as elements that escape from the grammatical domain to the pragmatic and communicative domain. This paper argues with the grammaticalization of DMs. It provides evidence to this view from Jordanian Arabic. The cases under investigation in this paper are the DMs *ma*, *hu* and *mahu*. This paper offers synchronic evidence supporting their grammaticalization. This evidence is based on two main conclusions: (1) these DMs share common grammatical items most of the grammaticalization sub-processes that lead to their emergence, and (2) they are not dis-integrated elements from the sentence grammar. They rather belong to the peripheral domain of grammar.

## 1 Introduction

As a type of language change, grammaticalization, for most researchers, is a cross-linguistic phenomenon heavily recurring in synthetic languages, and a cognitive tendency (see Traugott 2002 for further discussion of the onset and triggers of grammaticalization).^1^ It occurs in chains that may recur in natural languages. Grammaticalization forms grammar by the gradual shift from the lexical domain to the functional domain, such as the development of futurity-denoting modal verbs from motion verbs (Bybee et al., 1991). It may lead to expansion in functionality by acquiring new grammatical meanings (e.g., the ability-denoting modal verb *can* in English evolved further to mean possibility (Ziegeler 2011)), and phonological and morphosyntactic reduction to increase morphosyntactic bondedness, such as turning *will* into the clitic *’ll*.

In the last two decades, grammaticalization has been challenged. One of the main challenges to grammaticalization is the implementation of its approaches on discourse material, including discourse markers (henceforth DMs).^2^ Its approaches become hard-pressed when implemented on such material as they are typically syntactically and prosodically non-integrated and do not contribute to the propositional content of a sentence, as assumed by Heine (2018).^3^ They rather help in figuring out the speaker's attitude, regulating the discourse units and defining the relation between interlocutors (Fraser 1999). Hence, some linguists (e.g., Aijmer 1997; Günthner and Mutz 2004; Frank-Job 2006) have proposed that the evolution of DMs is a movement away from core grammar towards pragmaticalization, which is the evolution of context-level markers from grammatical or content words.

Some other researchers propose expanding the domain of grammar to encompass discourse material (for more details of this integration see Traugott 1995; Himmelmann 2004; Diewald, 2011a, Diewald, 2011b). Consequently, the evolution of DMs should be a form of grammaticalization. In this view, the concept *pragmaticalization* is not necessary to capture the development of DMs due to the following observations: (1) many grammatical markers have pragmatic extensions and (2) many DMs submit to morpho-syntactic and phonological processes, which are generally typical processes in grammar. Another view presents DMs as the output of two independent stages; grammaticalization of content words followed by pragmaticalization (see Erman and Kotsinas 1993; Onodera 1995; Marchello-Nizia 2009). They also indicate that some DMs can be a product of the direct pragmaticalization of content words. Hence, grammaticalization and pragmaticalization in this view are two distinct processes of language change, and only the latter is obligatory in the course of developing a DM.

More recently, Heine (2013, 2018) casts doubts on the evolution of DMs as cases of grammaticalization or pragmaticalization. He questions whether all the processes that are integral to grammaticalization are applicable or necessary to the emergence of DMs. For Heine, the emergence of DMs does not seem to be a prototypical case of grammaticalization. It is rather the direct and instant transition from the domain of semantics to that of pragmatics and/or discourse. In this view, DMs are developed as theticals by cooptation ‘whereby information units such as clauses, phrases, or words are transferred from the domain of sentence grammar to that of discourse organization’ (Heine 2013:1205).

From the previous discussion, it can be inferred that the development of DMs in a way analogous to that of common grammatical markers, such as auxiliary verbs, is controversial. This paper argues with the grammaticalization of DMs. It is to explore the emergence of three interrelated DMs in Jordanian Arabic (JA), namely, *hu*, *ma* and *mahu* and show that they are cases of grammaticalization. This proposal relies on two observations: these DMs underwent most of the prototypical mechanisms of grammaticalization, and they are not syntactically disintegrated from sentence grammar. They are rather visible to syntactic operations. In the current paper, a grammaticalization path to the development of these DMs in JA, based on their synchronic properties, is also proposed. The DMs *hu*, *ma* evolved from two grammatical morphemes (i.e., the third person masculine singular pronoun *hu:* and the negation particle *ma:*) and the DM *mahu* is a product of a non-trivial coalescence of the DMs *ma* and *hu*. The discourse and pragmatic functions of these DMs in JA are also investigated. Concerning their discourse function, they are discourse linkers that mark a turn as responsive in the oral discourse. On the other hand, at the pragmatic/attitudinal level each of these DMs has its own peculiarity.

The outline of the current paper is as follows: Section 2 is mainly to introduce the prototypical sub-processes of grammaticalization, and to offer an overview of studies that tackle grammaticalization in Arabic. Section 3 investigates the functions of the grammatical markers *hu: ma:*, their development into DMs and their discourse and pragmatic functions. It also explores the evolution and functions of the DM *mahu*. Section 4 and 5 are to argue with the grammaticalization of these DMs. Section 4 provides synchronic evidence that these DMs are characterized by some prototypical sub-processes of grammaticalization, whereas Section 5 is to show that these DMs should be subsumed under sentence grammar (i.e., they are not disintegrated elements).

## 2 Grammaticalization

In this section, the prototypical processes of grammaticalization are introduced and previous studies concerned with grammaticalization in Arabic are reviewed.

### 2.1 Grammaticalization processes

Grammaticalization is a multilayered process of language change that encompasses multiple semantic, morphosyntactic and phonological interacting processes that typically apply gradually (Lehmann's 1995 [1982]). It rests on four common internal mechanisms: desemanticization (or semantic bleaching), decategorialization (morphological reduction/loss), phonetic erosion (or reduction) and obligatorification (or obligatoriness). Desemanticization is the loss or reduction of the meaningful content of a word. Decategorialization, from a grammatical perspective, is a process by which an item from an open class (major category) is dropped into a closed class (minor category) (Hopper and Traugott, 1993). This results in losing characterizing properties. For example, when lexical verbs shift into the domain of auxiliaries, they will not be able to ‘form the predicate nucleus of the phrase and to take arguments’ (Heine and Kuteva 2002). Decategorialization is typically accompanied by the loss of inflection and syntactic reanalysis (see Heine 2003; Lehmann 2004; Heine and Kuteva 2007). Once a lexical verb is evolved into an auxiliary verb, it is syntactically raised to little v head and it is expected not to inflect the phi-features of its subject. In other words, its form undergoes freezing.

Following the desemanticization of a lexical word and the development of a new functional item, the functional item becomes prone to phonetic erosion. Phonetic erosion can be defined as the reduction of the phonological substance of the newly formed functional item in some way resulting in making it ‘more dependent on surrounding phonetic material’ (Heine 2003). The common view of this process is that it is a late step in the fabric of grammaticalization and is bound with frequency and predictability (Traugott 2002). This implies that the more frequent a functional item is, the more predictable and prone to be turned into a phonetically economical form it will be. A word may lose a segment, a syllable or a suprasegmental feature (e.g., stress), and this loss makes the target word contingent on one of the adjacent words. With regard to obligatorification, it can be defined as a mechanism by which a grammatical device becomes obligatory in a specific morpho-syntactic context. However, there is no consensus whether or not this mechanism is mandatory in grammaticalization, as a type of language change.

### 2.2 Overview of grammaticalisation in Arabic

Grammaticalization in Arabic is an understudied domain. However, there are a number of previous studies that tackle the issue of grammaticalization in Arabic vernaculars. The focus of some of these studies is the shift of some content verbs into the grammatical domain of future and aspect markers, and the diachronic paths of their grammaticalization. To mention some, Alshboul et al. (2010) investigate the development of the lexical verbs *ra:ħ* ‘went’ and *baddi* ‘I want’ to future markers in JA. Similarly, Jarad (2013) explores the grammaticalization of the motion verb *ra:ħ* as a future marker in Syrian Arabic. They report that this grammaticalization is paired with semantic, morphological, phonetic and morpho-phonological shift. Additionally, Jarad (2017) investigates the grammaticalization of a number of lexical items into grammatical ones in Emirati Arabic, including the grammaticalization of the volitional verb *ʔabɣi* ‘I want’ into a future marker. With regard to aspect markers, the development of some posture verbs to progressive aspect markers are studied in Kuwaiti Arabic (Al-Najjar, 1991), Emirati Arabic (Al-Najjar, 1991), Urban Hijazi Arabic (Basulaiman 2018) and vernacular Arabic, in general (Camilleri and Sadler 2017). Some other studies on grammaticalization focus on pronominal markers, prepositions, subordinators (see Wilmsen, 2017; Druel, 2010) and copulas derived from pronouns or posture verbs in Arabic (Camilleri and Sadler 2019).

With regard to DMs, the majority of the previous studies on DMs are concerned with their pragmatic functions and uses in daily conversations, such as the DM *ʕa:di* ‘normal’ (Kanakri and Al-Harahsheh 2013), *wallahi* ‘by Allah’ (Al-Khawaldeh 2018), *lakin* ‘but’ (Alsager et al., 2020) and *ya:hummala:li* (Hamdan and Abu Rumman, 2020).^4^ To the best of the researcher's knowledge, there are few studies that tackle the development of some DMs from lexical or functional sources, such as the grammaticalization of the wh-word *ʃuː* into a discourse linker Jarrah et al. (2019). However, investigating the grammaticalization and grammaticalization paths of DMs in Arabic remains an understudied area.

## 3 The grammaticalization of three DMs in Jordanian Arabic

In this section, the synchronic properties of the three DMs are investigated, and a path to their development is suggested. Further, it is argued that these DMs are evolved from grammatical items, namely, the third person masculine singular pronoun *hu:* and the negation particle *ma:*.

To address the issue of the development of the three target DMs in JA, the current study relies on the following sources: the intuition of the research as a native speaker of JA and naturally occurring data elicited from Twitter and Facebook free speech and JA television series. The dialect under scrutiny is the Jordanian dialect spoken in the following northern regions of Jordan: Irbid, Jerash, Ajloun and Al-Ramtha. For the sake of simplicity, the minimal phonological variations between the sub-varieties are ignored and neutralized while phonologically transcribing the collected data. The current study investigates the development of the three target DMs based on synchronic evidence. The diachronic aspect/evidence of the development of these DMs is beyond the scope of the current study, as the collected data are drawn from the current form of JA. Moreover, it is difficult to find written material and audio/visual recordings that document an older form of JA.

### 3.1 A discourse marker from a pronominal source

This part is to investigate the development of the DM *hu* from a pronominal source and suggest a path of its grammaticalization. It is also to discuss its discourse and pragmatic/attitudinal functions.

#### 3.1.1 The pronoun *hu:* as a copula and a discourse marker

The third person singular masculine pronoun *hu:* may appear in sentence-initial position as a subject or topic, as can be seen in (1a). It may also appear in a post-verbal position where it is typically under focus, as exemplified in (1b). (PARTICIPLE = PART).

**Table:** 

(1)	*a. hu:*	*(illi)*	*ka:n*	*wa:gif*	*hna:k*
	3SG.M.NOM	that	be.3SG.M.PST	stand.PART	there
	‘He was standing over there.’ or				
	‘He was the one standing over there.’				
	*b. smiʕ-t-u*	*hu:*			
	hear-1SG-3SG	3SG.M.NOM			
	Intended: ‘I heard him not anybody else.’				

As shown in (1a), the verb must agree with this pronoun in phi-features, akin to other pronouns. Any mismatch results in ungrammaticality. The cause of the ungrammaticality of the sentence in (2) is that the verb, which is marked with the feminine suffix -*at*, does not agree with the masculine pronominal subject *hu:* in gender.

**Table:** 

(2)	∗*hu:*	*ka:n-at*	*wa:gif-eh*	*hna:k*
	3SG.M.NOM	be.PST-3SG.F	stand.PART.SG.F	there
	Intended: ‘She was standing over there.’			

Additionally, *hu:* developed a grammatical function: it acts as a pronominal copula in present tense sentences in JA and in some other Arabic vernaculars, such as Egyptian Arabic (Eid 1983, 1991) and Lebanese Arabic (Choueiri 2016) (see Benmamoun 2000; Aoun et al., 2010; Alharbi 2017 for further discussion of the pronominal copula in Arabic).^5^ In (3), *hu:* functions as a copula.^6^ It links a definite complement to its subject in a predicational sentence in (3a) and the two DPs of an identificational/equational sentence in (3b). Based on language economy and the general observation that the masculine singular is the unmarked form in Arabic, the masculine singular *hu:* should be the only third person pronoun which has been developed into a copula. To account for its variants, this form inflects for number and gender to establish agreement with the phi-features of its subject, such as the feminine plural *hinne* in (3c). Hence, the copular *hu:* is strongly contingent on the host structure; that is to say, it must reflect the phi-features of the subject in equational and predicational sentences and occupies a fixed position in these sentences. (SUPERLATIVE = SUPER).

**Table:** 

(3)	a. *ʔil-binni*	*hu:*	*ʔaħsan*	*lu:n*	*la-ha:ð*^*ʕ*^a	*ʔil-ba:b*
	DEF-brown	COP.3.SG.M	good.SUPER	color	to-this	DEF-door
	‘The brown color is the best one to paint this door.’					
	b. *ħamu:deh*	*hu:*	*nafs-uh*	*ʔiħmad*		
	Hamudeh	COP.3.SG.M	Self-3.SG.M	Ahmad		
	‘Hamudeh is Ahmad.’					
	c*. haðu:l*	*ʔil-ʔalwa:n*	*hinne*	*ʔil-ʔaħsan*	*la-ha:ð*^*ʕ*^	*ʔil-ba:b*
	These.PL	DEF-colors	COP.3.PL.F	DEF-good.SUPER.	to-this.M	DEF-door
	‘The brown and the black are the best colors to paint this door.’					

It is worth noting here that the pronominal *hu:* is not the only copula that vernacular Arabic developed to substitute zero copula, especially in imperfective sentences. The active participle form *ga:ʕid* ‘sitting’ is another source to Arabic copulas. Camilleri and Sadler (2019) traced the emergence of this copula in some Arabic varieties. They reported that it is a fully-fledged copula that can express a locative or temporary sense in Maltese Arabic, whereas its use is still very restricted in use (i.e., it can only have a locative sense) in some other Arabic varieties, such as Kuwaiti and Hijazi Arabic. With regard to JA, *ga:ʕid*, similar to several Arabic dialects, can only serve as an aspectual marker denoting progression and developed no copular function, to the best of the researcher's knowledge as a native speaker of JA. This is, may be, because it seems much easier for the pronoun *hu:* to develop a linking function in predicational and equational sentences than for *ga:ʕid*, which has a locative sense.

What also argues with the development of the pronoun *hu:* into a copula lies in the way it establishes agreement with the subject when the subject-complement order is reversed. This may result in modifying the phi-features of the copular *hu:*. In (4a), the copula *hu:* agrees with the masculine singular subject *sabab*. Once the order of the singular topic and the plural complement is reversed, as shown in (4b), the copula is re-specified as plural to agree with *ʔil-dʒamahi:r*.

**Table:** 

(4)	a. *sabab*	*ʔil-fu:z*	*hu:*	*ʔil-dʒamahi:r*
	Reason	DEF-win	COP.3SG.M	DEF-crowds
	‘The cause of the win is the crowds.’			
	b. *ʔil-dʒamahi:r*	*hummu*	*sabab*	*ʔil-fu:z*
	DEF-crowds	COP.3PL.M	reason	DEF-win
	‘The crowds are the cause of the win.’			

A synchronic observation that stands with the evolution of a copula from the third person singular pronoun is that all Arabic varieties have the pronoun *hu:*, but some of them lack its copular counterpart, such as Modern Standard Arabic (Eid 1991). This observation implies that this grammaticalization path has not yet completed in some Arabic varieties.

The copular *hu:* can also function as an interrogative operator in JA. In (5), *hu:* is located at the left edge of the sentence to create a yes/no question.^7^

**Table:** 

(5)	*hu:*	*haða*	*ʕumar*?
	COP.3SG.M	This	Omer
	‘Is this Omer?’		

This initial position of the interrogative *hu:* licensed its development into a DM, as DMs typically precede the sentences they scope over. The importance of syntactic position in licensing grammaticalization was previously reported in the relevant literature. Jarrah's et al. (2019) proposed that ‘the syntactic position occupied by a given word is a key factor in deciding whether or not this word can be grammaticalized’. As can be observed in (6), *hu:* in initial position underwent phonological and morphosyntactic loss/reduction. It lost its stress, and then its long vowel underwent reduction. Further, it lost its ability to inflect: it cannot agree with the feminine subject *ʔil-fikrah*. Note that its presence is also optional, as marked by parentheses.

**Table:** 

(6)	(*hu*)	*ʔil-fikrah*	*ʔilli*	*ħaka-t-ha*	*ʔinn-u*
	DM	DEF-idea.F	that	speak.3SGF-3SGF.ACC	COMP-3SGM
	‘The idea that she suggested is that…’				

Given this optionality and its inability to have agreement in phi-features, the reduced *hu* in (6) is a DM that adds no semantic contribution to the proposition of the sentence over which it scopes.

#### 3.1.2 The functions of the discourse marker *hu*

At the discourse level, the DM *hu* acts as a discourse-linker in oral communications. It regulates the relation between turns. It marks an utterance it scopes over as non-initial (i.e., a responsive turn) and ties it to the ongoing conversation. In (7b), the turn starts with the DM *hu*. It operates over the entire utterance and links it to the utterance in the previous turn in (7a). On the other hand, in the absence of the DM *hu*, the utterance in (7b) can be either an initiative or responsive turn in the conversation. The DM *hu* can also link a responsive turn to a prior action/event that happened in the universe of the oral discourse. For exemplification, while the interlocutors in (7) are watching a lot of people rushing down the streets, Speaker 2 can react to this event, which is known for both interlocutors, with the utterance in (7b) even if Speaker 1 does not initiate the conversation. This entails that the DM *hu* marks an utterance as a response/reaction to former linguistic material or a previous event in the discourse universe.

**Table:** 

(7)	*(Context: Speaker 1 and Speaker 2 are standing on a window and talking about people rushing down the streets just right after the break of a curfew)*				
	a. Speaker 1:	*ʔil-na:s*	*zaj*	*ʔil-madʒan-i:n*	*b-il-ʃawa:riʕ*
		DEF-people	like	DEF-mad-PL.M	in-DEF-street.PL
		‘The people are down the street, just like a madding crowd’.			
	b. Speaker 2:	*hu*	*la:zim*	*n-inħadʒir*	
		DM	necessary	1PL-put in a quarantine	
		‘We should be put in a quarantine.’			

At the pragmatic/attitudinal level, the DM *hu* adds more emphasis on the proposition of the declarative sentence it scopes over when the sentence offers a different opinion from that in the previous turn. This function is common in the presence of a modal-like item in the sentence, such as the adjective *la:zim* ‘necessary’. In (8), the interlocutors are discussing the reaction of Speaker 1 to an event. Speaker 2 starts his turn with the DM *hu* to emphasize that the decision that has been made by Speaker 1 is wrong and spontaneous.

**Table:** 

(8)	*(Context: A person has just insulted Speaker 1. Speaker 1 reacted aggressively and is thinking of calling the police. Speaker 2 is trying to convince Speaker* 1 that *his reaction was spontaneous and he should not call the police.)*					
	Speaker 1:	*ʔana*	*ka:n*	*lazim*	*ʔaʃtaki*	*ʕale:-h*
		1SG	be.PST.3SG.M	must	complain.1SG	on-3SG.M
		‘I should have complained against him.’				
	Speaker 2:	*hu*	*ka:n*	*la:zim*	*tuskut*	
		DM	be.PST.3SG.M	must	stay silent.2SG.M	
		‘You should have stayed silent.’				

Besides, the DM *hu* with imperatives or interrogatives has a persuasive function. In (9), it occurs before the imperative *ʔuskut* to show that the speaker is insisting on stopping shouting. In (10), the DM *hu* to the left of the question indicates that this question has been repeated by the speaker, and the function of the DM is to persuade the hearer to answer.

**Table:** 

(9)	*(Context: a father is asking his little son to stop shouting for the second time.)*	
	*hu*	*ʔuskut*
	DM	stay silent.2SGM
	‘Shut up!’

**Table:** 

(10)	*(Context: One of the interlocutors is asking others to tell him the name of the person who informed them that is travelling soon.)*					
	*hu*	*mi:n*	*ʔilli*	*ħaka:-t-l-ak*	*haða*	*il-kala:m*?
	DM	who	that	speak.3SGF-to-2SG	this	DEF-speech
	‘Who (feminine) told you this?’					

Based on the previous discussion, the path that led to the emerging DM *hu* is proposed in (11). From the pronominal system in JA, the pronoun *hu:* was targeted to be developed into a copula and an interrogative operator, and then into a DM.

**Table:** 

(11)	Pronoun →	copula/interrogative operator →	DM
	*hu:*	*hu:*	*hu*

The development of the DM *hu* from the copula/interrogative operator and the development of the latter from a pronoun can be further supported by their synchronic properties. The common denominator among the pronominal, copular and discourse *hu*(*:*) is the linking function. The pronoun links its host sentence to a particular person or thing in the real world. Likewise, the copular *hu:* links two words or phrases (Brown and Miller 2013:112), namely, a subject and its complement in a predicational sentence or two DPs in an equational sentence. With regard to the DM *hu*, it links two turns, propositions or a turn and an action/event in the realm of the oral discourse. This entails that the linking function somehow persists throughout the grammaticalization path of the DM *hu*.

Worth highlighting here is that the other third person pronouns can be turn-initial; however, they have not been evolved into DMs. Evidence to their pronominal nature in this position is that the verb in the host sentence must establish agreement with the available third person pronoun. The sentence in (12) is ungrammatical as the verb *ħaka* cannot agree with the number feature of *hummu*.

**Table:** 

(12)	∗*hummu*	*mi:n*	*ʔilli*	*ħaka*
	3PL.M	who	that	say.PST.3SG.M
	‘Who just talked?’			

The next part is mainly to argue that the negative particle *ma:* evolved into a DM in JA.

### 3.2 The evolution of a discourse marker from a negation particle

In MSA, Arab grammarians sorted *ma:* into several types depending on its functions. It can be a interrogative particle, relativizer, negative particle, gerund producing, indefinite adjectival, indefinite exclamative or preventative. It can also be superfluous.^8^ In this section, the focus will be on *ma:* as a negation particle, which is common in JA, as exemplified in (13).

**Table:** 

(13)	*ma:*	*baddi*	*ʔadrus*
	NEG	want.1SG	study.1SG.IMPERF
	‘I do want to study.’		

Similar to *hu:*, *ma:* developed into a DM in JA. At the phonological level, *ma:* also underwent reduction when it was dropped in the domain of DMs, as shown in (14b). It lost its stress, and then its long vowel was reduced (shortened). At the discourse level, the DM *ma* is a discourse linker. It marks a turn as responsive. As can be observed in (14b), it links a responsive turn to a preceding one. To illustrate, Speaker 1 in (14a) is asking Speaker 2 to work with him; however, Speaker's 2 reply indicates that he may accept, but he needs to know what is the nature of that work first. It is worth noting here that the DM *ma*, similar to the DM *hu*, can link a responsive turn to a certain action or event in the universe of the oral discourse.

**Table:** 

(14)	*(Context: Speaker 1 and his friends have a project to finish. Speaker 1 is asking Speaker 2 to join the work team).*					
	a. Speaker 1:	*t-iʃtaɣil*	*maʕ-na*?			
		2-work.SGM	with-1PL			
		‘Would you like to work with us?’				
	b. Speaker 2:	*ma*	*badd-i*	*ʔaʕrif*	*ʃu:*	*il-ʃuɣul*
		DM	want-1SG	know.1SG	what	DEF-work
		*b-il-ʔawwal*				
		in-DEF-first				
		‘I need to know what is the nature of the work first.’				

In terms of distribution, the example in (14b) may indicate that the type of *ma:* that developed into a DM is the negation particle. To illustrate, the presence of the DM *ma* and the negative particle in the same position in (14b&15), should imply that the negation particle is the raw material of the DM *ma*.

**Table:** 

(15)	Speaker B:	*ma:*	*badd-i*	*ʔaʕrif*	*ʃu:*	*il-ʃuɣul*	*b-il-ʔawwal*
		NEG	want-1SG	know.1SG	what	DEF-work	in-DEF-first
		‘Do you think that it is not important to know the nature of the work first?’					

At the first sight, the negation particle *ma:* and its DM counterpart do not have shared features. More specifically, the negation particle contributes to the propositional content of the sentence, whereas the DM is semantically null and has a communicative function. However, what supports the view that the negation particle is the source of the DM is that the meaningful content of the negation particle was not fully bleached once it was developed into a DM. The negation meaning has persisted in the DM counterpart at the pragmatic level: the DM *ma*, in (15b), is basically to reject a previous proposition and replace it with that of the utterance over which *ma* operates. To illustrate, Speaker 2 rejects to work with Speaker 1 immediately. He wants to know more about the nature of this work first. Hence, the DM *ma*, which is a kin to *but* in English, is basically to express (temporary) refusal at the level of the oral discourse. Another example illustrating this pragmatic and communicative function is the one in (16). Speaker 2 disagrees with Speaker 1 who has suggested leaving to another workplace in the night shift. Speaker 2 refuses leaving the workplace as he and all his friends agreed to finish the work, and then they may go to another workplace.

**Table:** 

(16)	*(Context: a group of workers are working on a project on the day shift and are thinking of working on another project in the night shift).*					
	a. Speaker 1:	*n-iʃtaɣil*	*maʕ-hum*?			
		1PL.work.SGM	with-3PL.M			
		‘Should we work with them?’				
	b. Speaker 2:	*ma*	*badd-na*	*hassa*	*ʔin-xallis*^*ʕ*^	*haj*
		DM	want-1SG	now	1PL.finish	this
		*ʔil-warʃeh*	*b-il-ʔawwal*			
		DEF-workshop	in-DEF-first			
		Intended meaning: ‘We must finish this (our) task first.’				

Based on the previous discussion, the DM *ma* should be developed from the negation particle *ma:* and the negative connotation persists in the DM counterpart. The path of the DM *ma* is suggested in (17):

**Table:** 

(17)	Negative particle →	DM (discourse linker)
	*ma:*	*ma*

Below, the non-trivial coalescence of the DMs *hu* and *ma* is investigated.

### 3.3 The coalescence of two discourse markers

In JA, the DMs *ma* and *hu* were coalesced to form another DM, *mahu*. It should be highlighted first that this coalescence is not a case of two simple DMs superficially combined together only at the phonological level. It is rather a fully-fledged DM, as its components cannot be reversed or separated by an intervening element, as shown in the ungrammatical structures in (18).

**Table:** 

(18)	a. (*mahu*) (∗*huma*)	*ʔil-mat*^*ʕ*^*aʕi:m*,	*ʃu:*	*s*^*ʕ*^*a:r*	*fi:-hen*
	DM	DEF-vaccines	what	happened	in-them
	b. (∗*ma*)	*ʔil-mat*^*ʕ*^*aʕi:m* (∗*hu*),	*ʃu:*	*s*^*ʕ*^*a:r*	*fi:-hen*
	Literal translation: ‘The vaccines, what happened to them?’				
	‘The vaccines, are they ready?’				

At the discourse level, *mahu* links the utterance to the previous turn. At the pragmatic/attitudinal level, *mahu* is strongly persuasive, in comparison with the persuasive function of the DM *hu*. To clarify the persuading function of *mahu* and to compare it with that of the DM *hu*, consider the example in (19). In (20b), Speaker 2 wants to know the name of the person who told Speaker 1 that speaker 2 is traveling soon. Given the fact that it is the first time Speaker 2 requesting the name in (19b), he does not use any DMs. On the other hand, the use of the DM *hu* in (19d) indicates that Speaker 2 is repeating the same question after the ambiguous response of Speaker 1 in (19c). Speaker 2 is insisting one Speaker 1 this time to know the name of that person. Finally, Speaker 1 is trying his best to avoid mentioning that name in (19e), and the use of *mahu* in (19f) entails that Speaker 2 is over-insisting on his initial request and shows that he is too concerned. Hence, the DM *hu* has a persuading function, but *mahu* is strongly persuasive. As for the speaker's attitude, *hu* shows that he is concerned, whereas *mahu* denotes that he is too concerned and anxious.

**Table:** 

(19)	*(Context: Speaker 1 visits Speaker 2. Speaker 1 informs Speaker 2 that he has heard about Speaker's 2 intention to travel soon.)*				
	a. Speaker 1:	*smiʕit*	*ʔinna-k*	*badda-k*	*tsa:fir*
		Head.PST.1SG	COMP-2SG.M	want-2SG.M	travel.2SG.M
		‘I have heard that you will travel.’			
	b. Speaker 2:	*mi:n*	*ʔilli*	*ħaka:-la-k*?	
		who	that	speak.3SG.M-to-2SG.M	
		‘Who told you that?’			
	c. Speaker 1:	*smiʕit*	*min*	*ʔil-na:s*	
		hear.PST.1SG	from	DEF-people	
		‘I have heard that from people’			
	d. Speaker 2:	*hu*	*mi:n*	*ʔilli*	*ħaka:-la-k*?
		DM	who	that	speak.3SG.M-to-2SG.M
		‘Who told you that?’			
	e. Speaker 1:	*dagi:gah,*	*ʔarud*	*ʔala*	*ʔil-talifu:n*
		minute,	reply.1SG	on	DEF-phone
		‘One minute, I will answer the call.’			
	f. Speaker 2:	*mahu*	*mi:n*	*illi*	*ħaka:-la-k*
		DM	who	that	speak.3SG.M-to-2SG.M
		‘Tell me! Who told you that?’			

To conclude this section, the DMs *hu*, *ma* and *mahu* are discourse linkers with some variations in the attitudinal/pragmatic function(s). A grammaticalization path of these DMs is suggested in (20).

**Table:** 

(20)	Pronoun (*hu:*) → Copula	→	DM *hu*↘	Coalesced DM *mahu*
	Negation particle (*ma:*)	→	DM *ma*↗	

## 4 The evolution of DMs in JA

As introduced in Section 1, the extension of the domain of grammaticalization to cover the evolution of DMs is controversial as they shift from referential or grammatical meaning to metatextual, interpersonal and attitudinal meaning (Aijmer 1997; Diewald, 2011a, Diewald, 2011b). Therefore, it is viewed by some researchers as an escapee from the domain of core grammar, that is, they do not fully tolerate Lehmann's (1995 [1982]) criteria of grammaticalization as they do not undergo scope narrowing and obligatorification. They undergo expansion in scope as they no longer operate within the sentence-level. They rather scope over discourse units. Further, they are optional. However, some other researchers (e.g., Traugott 1995) propose that the domain of grammar and grammaticalization should be expanded in a way to cover the emergence of DMs. In this view, increase in scope and lack of obligatorification do not expel DMs from the realm of grammaticalization.

This section is to argue that the three target DMs in JA are cases of grammaticalization. What supports their grammaticalization is that they underwent some prototypical sub-processes of grammaticalization: desemanticization and phonetic erosion.^9^ At the semantic level, the copular *hu:*, which is specified as third person masculine singular, lost its phi-features once it was evolved into the DM *hu*, thus the DM *hu* does not refer to a particular person or thing and does not have agreement with any element in the sentence it scopes over. This type of semantic loss is common when typical grammatical(ized) items undergo further grammaticalization (so-called secondary grammaticalization). They tend to lose the ability to inflect (Heine 2003; Lehmann 2004; Heine and Kuteva 2007). Similar to *hu:*, the negation particle *ma:* lost some of its semantic content once it was evolved into a DM. However, the persistence of the function and the maintenance of some semantic content of an item in the DM counterpart is another observation that stands with the grammaticalization view of the target DMs in JA. This persistence is seen in (14b) where the DM *ma* that evolved from the negative particle *ma:*. In this example, the DM *ma* involves a negative connotation (i.e., the indirect refusal to a previous proposal in the discourse). Additionally, the linking function of the copular *hu* remains after its evolution to a DM and covers a communicative meaning. More specifically, the copular *hu:* and its DM counterpart have a linking function; however, the former links a complement to its subject and the latter links an utterance to the ongoing discourse. This implies that the functions of these DMs, albeit pragmatic, attitudinal and communicative, signify their development from other items.

With regard to phonetic erosion (reduction or attrition), the evolution of the copular *hu:* and the negation particle *ma:* into DMs is accompanied by the loss of some of their phonetic material (i.e., the loss of stress and reduction of its long vowel). Worth noting is that the disyllabic coalesced DM *mahu* can be reduced into monosyllabic *mu* in some JA sub-varieties.

Another important point that supports the view that the development of the three DMs are cases of grammaticalization is paradigmaticity, which is a process previously associated with grammaticalization (Lehmann 1995 [1982]). Paradigmaticity is characterized with having an oppositional pair or set. When an item is paradigmatized, ‘it builds an oppositional pair with another element and, in virtue of this, is a member of a paradigm.’ (Diewald 2011b: 367). With regard to the three DMs in the current paper, the presence of one of these DMs in a turn creates a paradigmatic opposition, that is, when a DM is scoping over an utterance to mark it as a responsive turn, the interlocutors will take it for granted that the DM-less counterpart is an initiative turn. However, this paradigmaticity is not fully restrictive as the opposite scenario is not true: the DM-less utterance can also occur when the turn is responsive. On this basis, the paradigmaticity of DMs is different from that of other grammaticalized items, such as copulas. The paradigmatic member of a DM is determined by the communicative intentions of speakers, whereas that of a copula is determined by language internal features (Diewald 2011b:368).

A point that may ostensibly argue against the grammaticalization of DMs is related to the stage of obligatorification (or obligatoriness), which is introduced by Lehmann (1995 [1982]). Obligatorification is not a feature of the three DMs in JA, unlike many grammatical markers. These DMs can be omitted without rendering the sentence it scopes over ungrammatical. However, this is not unobservable in core grammar. For exemplification, the deletion of the copular *hu:*, which should belong to core grammar, in a descriptive sentence in JA will not cause ungrammaticality of the host sentence, as can be observed in (21) below. Hence, obligatorification is not mandatory not only in the development of DMs, but also while forming some other grammatical items. This emphasizes on Lehmann's (1995 [1982]) view that the application of obligatorification is context-specific or language-specific.

**Table:** 

(21)	*ʔil-binni*	(*hu:*)	*ʔaħsan*	*lu:n*	*la-ha:ð*^*ʕ*^	*ʔil-ba:b*
	DEF-brown	COPULA	good.SUPER	color	to-this.M	DEF-door
	‘The brown color is the best one to paint this door.’					

Obligatorification arises once the copula or a DM surfaces in a sentence/utterance nonetheless. It is true that the (non-)presence of the copular *hu:* is not rule-based (not governed by grammar); however, when it is present, grammar obligatorily sets its inflectional morphology (i.e., number and gender). Similarly, the DM counterpart is not conditioned by inflectional grammar (it loses inflectional dependency and becomes fixed); however, its syntactic position in the turn is governed by rules: *ma* and *hu*, for example, are not allowed to occupy a sentence/turn-final position, whereas the DM *mahu* is allowed to appear in this position.

## 5 Discourse markers and sentence grammar

The aim of this section is to provide syntactic evidence to the integration of the three target DMs in sentence grammar. More specifically, they can interact with grammar-peripheral elements. In addition, the impact of this interaction on the information status of a host utterance is investigated.

Starting with the DM *hu*, it has been shown in Section 3 that it is optional and cannot inflect; however, it should not be considered a syntactically non-integrated (agrammatical) element. It is rather an element located at the left periphery of sentence grammar, due to its interactions with peripheral elements. The DM *hu* can precede or follow peripheral materials, such as ‘style disjuncts’ following Greenbaum's (1969) terminology, and topicalized elements. The DM *hu* can either precede or follow the peripheral style disjunct *badd-ak ʔil-s*^*ʕ*^*ara:ħah* in (22a) and the topic *ʔil-mat*^*ʕ*^*aʕi:m* in (22b). The DM *hu* cannot appear between the integral elements of a sentence and in final position when these elements are kept in situ, as marked by an asterisk in (22). This implies that this DM is not an integral element in sentence grammar; however, it is visible to syntactic operations (e.g., syntactic movement to a peripheral position (topic position)).

**Table:** 

(22)	a. (*hu*)	*badd-ak*	*ʔil-s*^*ʕ*^*ara:ħah,*	(*hu*)	*la:zim*	(∗*hu*)
	DM	want-2DGM	DEF-frankness	DM	must	DM
	*ħadʒaru:-na*		(∗*hu*)			
	1PL-IMPERF.quarantine		DM			
	‘To be frank, we should be put in a quarantine.’					

	b. (*hu*)	*ʔil-mat*^*ʕ*^*aʕi:m*, (*hu*)	*ʃu:*	(∗*hu*)	*s*^*ʕ*^*a:r*	(∗*hu*)
	DM	DEF-vaccines, DM	what	DM	happen.PST	DM
	*fi:-hen*	(∗*hu*)				
	in-3PLF	DM				
	Literal translation: ‘The vaccines, what happened to them?’					
	‘The vaccines, are they ready?’					

Similarly, the DM *ma* should be integrated in sentence grammar due to its clitic nature. Specifically, it can combine with a referring pronoun from the target sentence, such as the first person singular pronominal clitic -*ni* in (23). Note that the pronoun combined with *ma* must enter in agreement with one of the elements in the sentence in phi-features. A subsequent process to this combination is that a constituent from the sentence grammar can move up in the syntactic structure above this DM, such as *ʔil-ʃarikah* in (24b). It does not go without mentioning that the entire sentence can appear to the left of *ma-hi*, as in (24c). This indicates that it has been moved up in the syntactic representation above the DM *ma-hi*. Note that the combination of the DM *ma* with a referring pronoun (a part of the sentence grammar) licenses its presence in any position in a sentence even in the absence of any movement in the syntactic structure, as marked between parentheses in (24).

**Table:** 

(23)	a. *ma-ni*	*badd-i*	*ʔa-ħki*
	DM-1SG	want-1SG	1SG-talk
	‘I want to talk.’

**Table:** 

(24)	a. (*ma-hi*)	*ʔil-ʃarikah*	*msakreh*		*hassa*	
	(DM-3SGM)	DEF-company	closed		now	
	‘The company (the bill desk) is closed now.’					
	b. *ʔil-ʃarikah*,	(*ma-hi*)	*msakreh*	*hassa*		
	c. *ʔil-ʃarikah*	*msakreh*	*hassa,*	(*ma-hi*)		
	d. (*ma-hi*)	*ʔil-ʃarikah*	(*ma-hi*)	*msakreh*	(*ma-hi*)	*hassa* (*ma-hi*)

With regard to the DM *mahu*, comparable to the previous DMs, it can appear to the right or left edge of peripheral elements, such as style disjuncts or moved constituents. Hence, it can appear in various positions in an utterance: in utterance-initial position in (25a), to the right of a moved constituent in (25b) and in utterance-final position in (25c) after moving the entire sentence above this DM in the syntactic structure. However, it cannot be located between the integral elements of the sentence, which are in situ, as marked in (25d).

**Table:** 

(25)	a. (*mahu*) *ʔil-ʃarikah*	*msakreh*	*hassa*
	b. *ʔil-ʃarikah*, (*mahu*)	*msakreh*	*hassa*
	c. *ʔil-ʃarikahmsakreh*	*hassa*	(*mahu*)
	d. *ʔil-ʃarikah* (∗*mahu*)	*msakreh*	(∗*mahu*) *hassa*

To wrap up, it has been shown that the three DMs can be accounted for in sentence grammar. They are visible to syntactic movement, and therefore they should belong to the domain of peripheral grammar. Note that the DM *ma* has a clitic nature, and consequently it may appear in various position in sentence grammar.

In addition to the interaction of these DMs with peripheral elements in sentence grammar, the position of a DM can have an impact on the information content of the host utterance. Starting with the DM *mahu*, its presence early indicates that the information content of the host utterance is new, whereas in final position it is given. Consider the example in (26) where *mahu* in Speaker's 2 turn-initial position is used to presuppose that Speaker 1 does not know this information. On the other hand, its presence in turn-final position signifies that Speaker 2 presupposes that Speaker 1 knew this information about his car, and therefore it can be understood from this turn that Speaker 2 is blaming Speaker 1 for his carelessness.

**Table:** 

(26)	a. Speaker 1:	*ze:t*	*ʔil-sajja:rah*	*lu:n-uh*	*iswad*		
		Oil	DEF-car	color-3SG.M.POSS	black		
		‘The color of the engine oil is black.’					
	b. Speaker 2:	(*mahu*)	*la:zim*	*jitɣajjar*	*kull*	*ʃahur*	(*mahu*)
		DM	necessary	PST.change.3SG.M	every	month	DM
		‘It must be changed every month.’					

Similar conclusions can be said about the DM *ma* combined with a referring pronoun. In the presence of *mahinne* in turn-initial position in (27b), the information content is new, whereas in turn-final position, it is old.

**Table:** 

(27)	a. Speaker 1:	*ʔil-sajjarah*	*ma:*	*waggaf-at*	*maʕ-i*	*bidʒu:z*	*bre:k-a:t*
		DEF-car	NEG.	stopped-3SGM	with-1SG	possible	brake-PL.F
		‘The car did not stop. It is possibly the brakes.’					
	b. Speaker 2:	(*mahu*)	*l-bre:k-a:t*	*lazim*	*jitbaddal-in*	*ʔawwal*	
		DM	DEF-brake-PLF	must	PST.change-PL.F	first	
		*ʔil-ʃatwijjeh*	(*mahu*)				
		DEF-winter					
		‘The brakes must be changed once the winter season starts.’					

With regard to the DM *hu*, it does not appear in turn-final position. In this position, short forms are lengthened. From a prosodic perspective, this position is typically marked with utterance-final lengthening (i.e., the effect of the right boundary of the utterance domain especially on the word immediately at its left). The presence of the DM *hu* in this position will result in recovering the full form of its source material (i.e., the pronominal/the copular *hu:*) by vowel lengthening, as shown in (28). Hence, the ambiguity whether this item is a pronoun/copula or a DM in utterance-final position, prevents the occurrence of the DM *hu* in this position.

**Table:** 

(28)	(*hu*)	*la:zim*	*ħadʒaru:-na*	(∗*hu:*)
	DM	must	1PL-IMPERF.quarantine	DM
	‘We should be put in a quarantine.’			

As for the DM *ma*, it has a clitic nature and should not be stranded alone in utterance-final position. Further, its lengthening into *ma:* in this position causes the same confusion/ambiguity of leaving the DM *hu* in final position.

Depending on the previous discussion, although the three DMs in JA are optional and have metatextual and pragmatic functions, they should not be considered syntactically non-integrated elements as their development is characterized with the most common processes of grammaticalization and they can be accounted for within sentence grammar (i.e., they are visible to syntactic movement). Hence, the three DMs in JA should be subsumed within the realm of grammar and grammaticalization.

## 6 Conclusion

The current paper has argued with the view that the development of DMs should be studied as cases of grammaticalization, as previously proposed in (Traugott 1995; Diewald 2011b). In this paper, it was proposed that the domain of grammaticalization should encompass the development of the target three DMs in JA, namely, *hu*, *ma* and *mahu* for the following reasons. First, their development is indiscernible from the proper grammaticalization processes. It involves desemanticization, phonetic erosion and paradigmatization. Second, they are not disintegrated elements from sentence grammar. More precisely, they should be considered paraphrial elements within sentence grammar, as they are visible to syntactic operations (e.g., movement). Thus, they belong to the domain of peripheral grammar. Third, they influence the information structure of a host sentence. Hence, data from JA cast doubts on the proposal that the grammaticalization of DMs can be visualized as an escape from the domain of grammar, either core or peripheral. They rather argue with maintaining the development of DMs within the realm of grammaticalization.

## Declarations

### Author contribution statement

Abdulazeez Ahmad Jaradat: Conceived and designed the analysis; Analyzed and interpreted the data; Contributed analysis tools or data; Wrote the paper.

### Funding statement

This research did not receive any specific grant from funding agencies in the public, commercial, or not-for-profit sectors.

### Data availability statement

Data included in article/supplementary material/referenced in article.

### Declaration of interests statement

The authors declare no conflict of interest.

### Additional information

No additional information is available for this paper.

## Footnotes

1 In the relevant literature, *grammaticization* and *grammatization* are also introduced to refer to the same phenomenon.

2 Another major challenge of grammaticalization is steered toward the hypothesis of unidirectionality, which rules out degrammaticalized or lexicalized of grammatical items. Albeit rare, instances of degrammaticalization have been reported in some previous studies (Janda 2001; Willis 2007; Norde, 2009).

3 DMs are commonly flavored with some pragmatic/attitudinal meaning. They are typically short, procedural, optional and turn-initial.

4 The DM *ja:hummala:li* can be divided into three words: the vocative particle *ja:,* the pronoun *humm* and *la:li* ‘a lot of noise’ (Hamdan and Abu Rumman 2020).

5 Vernacular Arabic developed copulas of a four-way split: (a) the verbal copular: *ka:n* ‘be.PST. 3SGM’. (b) the zero copular. (c) the pronominal copula *hu:* ‘3SGM’. (d) the grammaticalized active participle form of the verb of posture *gaʕad* ‘sit’. Discussions on the contextual uses of each copula in vernacular Arabic can be found in Ryding (2005), Benmamoun (2008), Aoun et al. (2010), Alotaibi (2017) and (Camilleri and Sadler 2019).

6 Pronouns, beside deictic particles and verbs, are a common source to copulas in natural languages (Pustet 2003).

7 *hu:* in combination with the conjunction *w*- (or its variant *ʔu*-) ‘and’ behaves as an adverb of aspect denoting progression:

**Table:**

a. *ʃuft-uh*	*ʔu**-hu:*	*wa:gif*	*ʕal-ʔiʃa:rah*
See.PST.1SG-3SGM	CONJ-3SGM	stand.GERUND	on-street
‘I saw him while he was standing down the street.’			

8 In JA, the interrogative *ma:* normally combines with the reduced preposition -*l* ‘to’ to mean ‘what happened?’, as shown in (a). Note that the common operator in JA is *ʃu:* ‘what’. The adverbial *ma:* also exists in JA but it must be followed by the verb *da:m* ‘continues’, as shown in (b). *ma:* can also accompany a verb to form a gerund phrase as can be observed in the literal translation in (c) or functions as an exclamatory particle, as in (d). The relativizing *ma:* in JA must be preceded by *ʃu:*, as in (e). As for the superfluous, the indefinite adjectival and the preventive *ma:*, they are not observed in JA.

**Table:**

a. *ma:-l*	*ʔab-u:k*		
What-to	father-2SGM		
‘What happed to your father?’			
b. *ma*	∗(*da:mn-i*)	*ʕa:jiʃ*	
ADV	continue-1SG	alive.	
‘As long as I am alive.’			
c. *ʔixlafit*	*baʕid ma:*		*waʕadit-ni*
violate.PST.3SGM	after	GERENDIVE	promise.PST.3SG-1SG.ACC
Literal: ‘You violated after promising me’.			
‘You did not keep promise’			
d. *ma:*	*ʔaħla*	*h-al-be:t*	
EXCL	beautiful.COMP	DEM-DEF-house	
‘What a beautiful house!’			
e. *ʔiʕmal* ∗(*ʃu:*)	*∗*(*ma*:)	*badd-ak*	
Do.2SGM what	REL	want-2SGM	
‘Do whatever you like.’			

9 The evolution of the three DMs in JA should involve early decategorialization (i.e., changing the lexical status of a certain word (an open class) into a pronominal grammatical status). Diachronically and synchronically, it is too difficult to identify that lexical item.
